# Purpura Rheumatica

**Published:** 1909-01-02

**Authors:** 


### Diseases of Children.

### PURPURA RHEUMATICA.

One of the purpuric affections has been described under the names of purpura rheumatica, peliosis rheumatica, and Schönlein's disease. This variety of nomenclature is indicative of ignorance of its ætiology, though the addition of the qualifying adjective suggests a rheumatic causation. It is worth while to refer to two clinical cases illustrative of the characteristic features.

A boy, eight years old, was admitted to hospital under the writer's care with an indefinite history of a few days' duration. There had been some gastric and intestinal symptoms, and probably a mild degree of fever, before the appearance of a purpuric rash on the lower limbs and buttocks and pains in some of the joints, with a little swelling of one knee and ankle. He appeared to be in good health except for these symptoms, and had a fair appetite and little fever. In the course of a few days the symptoms subsided. Then, after the lapse of a few more days he suddenly developed œdema of the penis, especially the prepuce, followed by hæmorrhagic staining, and later on fresh purpuric spots and bruises. These signs cleared up in another week, and he was allowed up: fresh spots then came out on the lower limbs, and again cleared up after a few more days in bed.

A somewhat similar case was described by Ibotson (*Lancet*, 1906, ii. 159) in a boy aged two years. He had an œdematous prepuce, purpuric spots on the thighs, and no fever. Next day the temperature rose to 100° F., and both urticarial and purpuric spots came out on the extremities. Later on he had spots on the face, tenderness of the bones, and swelling of the left knee. He got well.

The prodromata of this variety of purpura include anæmia, languor, vague pains, vomiting or diarrhœa, and sometimes urticaria. Then the temperature rises, commonly about two degrees, but perhaps as much as five or six degrees. The rash is generally on the lower limbs and buttocks and on the forearms. Less often it is scattered all over the body and limbs, and even the face. Sometimes it resembles an urticaria with purpura in the centre of the wheals, and occasionally it is associated with erythema nodosum or erythema multiforme exudativum. The purpuric parts may be œdematous, secondarily to the purpuric rash. Thus œdema of the legs is not uncommon, particularly in patients allowed out of bed too soon. More often the œdema precedes the purpura, or occurs independently of it in the eyelids, face, penis, scrotum, labia, etc. Joint pains are common, and joint swelling is not infrequent. The ankles and knees are the most liable, and are affected with a peri-articular rather than an intra-articular effusion. Occasionally hæmorrhages take place from the mucous membranes—*e.g.* the nose and bowels. Intestinal bleeding may be associated with colic and vomiting, very suggestive of a stage of Henoch's purpura. The spleen may be enlarged.

The prognosis is excellent. Many patients are well in a couple of weeks. Others relapse, with rash, fever, and articular swelling, and exhibit a more or less paroxysmal course, sometimes of many weeks' duration, but ending in recovery. The heart is not involved, and there is rarely any albuminuria.

A consideration of the above description and cases indicates that the different varieties of purpura known under the names of simplex, rheumatica, hæmorrhagica, fulminans, and Henoch's purpura, are merely different degrees of the same affection; that they are not rheumatic in origin; that they are allied to urticaria and similar affections. The primary cause is probably a blood change of a toxic nature. In support of this one may refer to the fact that the spleen is frequently enlarged and that purpuric spots are apt to appear during many toxic and infective states. Purpura convalescentium occurs during convalescence from scarlet fever, measles, and, less often, diphtheria. Fatal cases have ensued on scarlet fever. In one of these, a boy in his fourth year (Biss, *Lancet*, 1902, ii. 284), the rash developed on the eighteenth day with oozing from the gums, hæmatemesis, and melæna, ending fatally in 36 hours. The changes in the kidneys indicated an acute toxæmia. There was no sign of nephritis during life, but after death the kidneys showed an acute degeneration which had converted them almost entirely into fat. An intensely acute nephritis has also been reported in cases of the Henoch type. Cachectic states of the system give rise to purpura through altered blood and tissue nutrition. Thus purpuric extravasation may appear in marasmic states from all causes, in chronic renal affections, and in various blood affections. It may be produced by drugs, by snake-venom, and by other causes. Hence we may sum up our knowledge of its ætiology by putting forward the view that it is due to blood changes which permit of extravasation, and that the blood change is produced by toxins and poisons of various sorts, marasmus, or special blood affections. The evidence is in favour of a gastro-intestinal origin of the poison, but there is no doubt that in some instances infection makes its entry through the tonsils or fauces, and possibly it may occasionally be rheumatic in character.

Essential purpura occurs equally in either sex and at all ages, though rare under two years. Various observations have been made on the blood. There have been noted reduction in specific gravity and percentage of hæmoglobin, slowing or absence of coagulation, diminution in the number of red cells and blood platelets, the appearance of megaloblasts and microblasts, an occasional myelocyte, and leucopenia, except perhaps at the onset. Leucocytosis has been noted in purpura hæmorrhagica and in Henoch's purpura, but no organisms have been found. Leucocytosis is in favour of secondary purpura. Prolonged cases of the hæmorrhagic variety are much like scurvy, and the blood resembles that of scurvy. Leucocytosis is very inconstant, whereas it is common in rheumatic fever. Auto-intoxication with a vaso-motor toxin is indicated by its resemblance to erythema multiforme associated with urticaria. One warning should be given as to diagnosis. In rare instances cerebro-spinal meningitis starts with a purpuric eruption like simple purpura, but the child is much more ill.

For the simple variety under discussion rest in bed, warmth, and suitable diet are often sufficient to bring about a rapid cure. A few doses of calcium lactate should be given. Calomel is valuable as an intestinal antiseptic, and it is advisable to clear out the bowel at the onset of the affection. Salicylates are of doubtful advantage, but may be given tentatively. Iron and arsenic are needed in convalescence, and it is of importance not to allow the child to get up until the symptoms have subsided for quite a week.

